# Supplementary material for: Identification of photoperiod-induced specific miRNAs in the adrenal glands of Sunite sheep (Ovis aries)
Source: Front Vet Sci. 2022 Jul 22;9:888207. doi: 10.3389/fvets.2022.888207 (PMC9354845; doi:10.3389/fvets.2022.888207)
Supplement: Supplementary file 1 [file Data_Sheet_1.ZIP › Supplementary file/miRDeep2 file.zip!/miRDeep2 file/Know_Structure/SPLP42A2/oar-mir-433.pdf]

miRBase precursor : oar-mir-433
Total read count : 1560
oar-miR-433-5p read count : 1
oar-miR-433-3p read count : 1559
remaining reads : 0

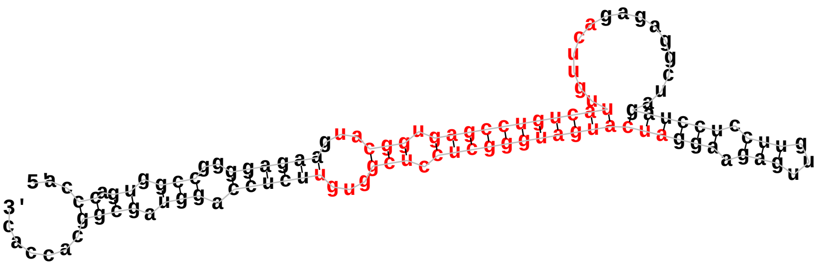

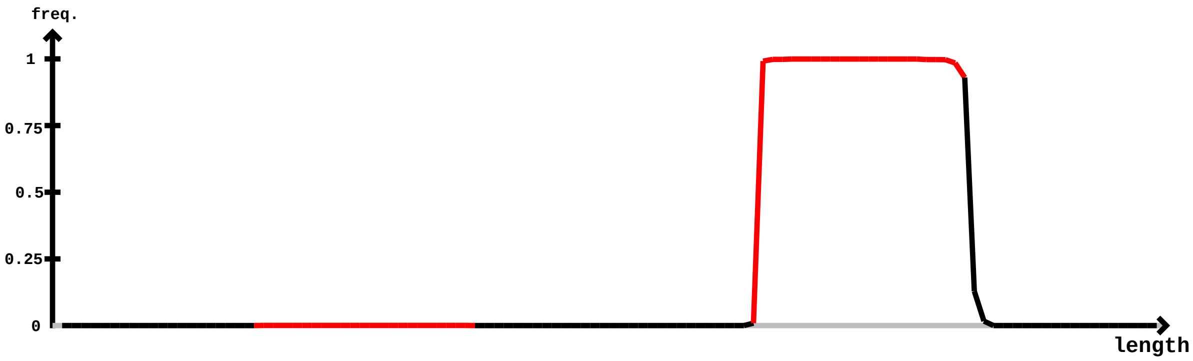

oar-miR-433-5p

oar-miR-433-3p

```
5'- acccaguggccggggagaaguacggugagccugucauuguucagagaggcuagauccuccuuguugagaaggaucaugaugggcuccucggugucuccagguagcggcaccac   -3'    exp
    ..((.((.(((..((((((...(((.(((((((((((..............((((((.(((...))).))))))))))))))))).)))...)))))).))).))))......   reads      mm         sample
    ....................uacggugagccugucauuguuc........................................................................   1          0          seq
    .......................................................................gaucaugaugggcuccucggug.....................   3          0          seq
    .......................................................................gaucaugaugggcuccucggugu....................   11         0          seq
    ........................................................................aucaugaugggcuccuc.........................   3          0          seq
    ........................................................................aucaugaugggcuccucgg.......................   1          0          seq
    ........................................................................aucaugaugggcuccucggu......................   19         0          seq
    ........................................................................aucaugaugggcuccucggug.....................   82         0          seq
    ........................................................................aucaugaugggcuccucggugu....................   1227       0          seq
    ........................................................................aucaugaugggcuccucgguguu...................   175        0          seq
    ........................................................................aucaugaugggcuccucgguguuc..................   26         0          seq
    .........................................................................ucaugaugggcuccucggugu....................   10         0          seq
    ...........................................................................augaugggcuccucggugu....................   2          0          seq
```
